# Mitochondrial Dysfunction and Acute Fatty Liver of Pregnancy

**DOI:** 10.3390/ijms23073595

**Published:** 2022-03-25

**Authors:** Raghu Ramanathan, Jamal A. Ibdah

**Affiliations:** 1 Division of Gastroenterology and Hepatology, University of Missouri, Columbia, MO 65211, USA; raghu.ramanathan@health.missouri.edu; 2 Harry S. Truman Memorial Veterans Medical Center, University of Missouri, Columbia, MO 65201, USA; 3 Department of Medical Pharmacology and Physiology, University of Missouri, Columbia, MO 65211, USA

**Keywords:** liver, mitochondrial dysfunction, β-oxidation, mitochondrial trifunctional protein, long chain 3-hydroxyacyl Co-A, acute fatty liver of pregnancy

## Abstract

The liver is one of the richest organs in mitochondria, serving as a hub for key metabolic pathways such as β-oxidation, the tricarboxylic acid (TCA) cycle, ketogenesis, respiratory activity, and adenosine triphosphate (ATP) synthesis, all of which provide metabolic energy for the entire body. Mitochondrial dysfunction has been linked to subcellular organelle dysfunction in liver diseases, particularly fatty liver disease. Acute fatty liver of pregnancy (AFLP) is a life-threatening liver disorder unique to pregnancy, which can result in serious maternal and fetal complications, including death. Pregnant mothers with this disease require early detection, prompt delivery, and supportive maternal care. AFLP was considered a mysterious illness and though its pathogenesis has not been fully elucidated, molecular research over the past two decades has linked AFLP to mitochondrial dysfunction and defects in fetal fatty-acid oxidation (FAO). Due to deficient placental and fetal FAO, harmful 3-hydroxy fatty acid metabolites accumulate in the maternal circulation, causing oxidative stress and microvesicular fatty infiltration of the liver, resulting in AFLP. In this review, we provide an overview of AFLP and mitochondrial FAO followed by discussion of how altered mitochondrial function plays an important role in the pathogenesis of AFLP.

## 1. Introduction

The liver is a vital organ that regulates various metabolic processes such as carbohydrate, lipid, and protein metabolism. It consists of various cell types (such as endothelial and epithelial cells, Kupffer cells, and stellate and Ito cells), with hepatocytes accounting for 70–85% of the total organ [1]. The liver is the hub of several intermediate metabolic pathways, including anabolic pathways that synthesize glucose, lipids, and ketones to meet the body’s energy demands. It is also one of the richest organs in mitochondria, and hence, it is an important site for key metabolic mitochondrial pathways such as the β-oxidation cycle, the tricarboxylic acid (TCA) cycle, ketogenesis, respiratory activity, and adenosine triphosphate (ATP) synthesis, which provide metabolic energy for the body as a whole [2].

The liver undergoes continuous adaptation or “remodeling” of mitochondrial energetics, gene expression, and morphology in response to increased metabolic demand, which plays a key role in the pathogenesis of liver disease [3,4]. Mitochondrial dysfunction has been linked to subcellular organelle dysfunction in liver diseases, especially fatty liver disorders [5]. Mitochondrial dysfunction is linked to the development of reactive oxygen species (ROS), and subsequent liver exposure to oxidative stress leads to inflammation and fibrosis [6,7].

Acute fatty liver of pregnancy (AFLP) is an uncommon condition characterized by microvesicular fatty infiltration in the liver [8]. AFLP remains a life-threatening condition in the third trimester of pregnancy with a high mortality rate [9]. Although the etiology of AFLP was long considered mysterious, ample evidence has connected mitochondrial abnormalities and impaired fatty-acid oxidation to AFLP.

In this review article, we discuss the role of mitochondria and their dysfunction as an underlying etiology for AFLP. First, we provide an overview of AFLP, the mitochondrion, its biogenesis, mitophagy, and metabolic function followed by a discussion of the role of mitochondrial dysfunction in the development of AFLP.

## 2. Acute Fatty Liver of Pregnancy (AFLP)

AFLP is a rare, third-trimester liver disorder unique to pregnancy with significant perinatal and maternal mortality. It was first described in 1934 by Stander as “yellow acute atrophy of the liver”. The incidence of AFLP is reported to be from 1 in 7270 to 1 in 13,000. The disease tends to recur in future pregnancies in approximately 20% of pregnancies complicated by AFLP. The underlying molecular mechanism for this recurrence is likely the strong association between maternal AFLP and pediatric fatty-acid oxidation defects [10] as described in detail in Section 4 of this review.

### 2.1. Clinical Presentation

AFLP presents in the third trimester, rarely late in the second trimester. The initial presentation is typically vague and include general symptoms such as epigastric pain, nausea, and vomiting [10] associated with elevation in liver transaminases. Occasionally, AFLP may present as asymptomatic elevation of transaminases, and jaundice is seen on initial presentation in severe cases [11]. Rarely, AFLP may present with symptoms of acute liver failure including encephalopathy and bleeding due to coagulopathy, but typically these symptoms are seen 1–2 weeks after initial presentation. AFLP is associated with preeclampsia is in >50% of cases.

### 2.2. Complications

Hypoglycemia, acute pancreatitis, infection, and acute renal failure are among the early complications of AFLP, while hepatic encephalopathy is considered a late complication and suggests development of acute liver failure. These complications are associated with significant perinatal and maternal mortality. Delivery can be complicated with severe postpartum hemorrhage. Diabetes insipidus has been reported to complicate AFLP [11].

### 2.3. Diagnosis

AFLP is primarily diagnosed based on clinical criteria. Laboratory abnormalities include moderate elevation of liver transaminases. The severity of liver dysfunction does not correlate with the degree of transaminases elevation. Leukocytosis is often seen as well. Platelet count is typically normal except when disseminated intravascular coagulation (DIC) is present. Blood urea nitrogen (BUN) and creatinine are also generally elevated. As the disease progresses and liver function worsens, hypoglycemia, and encephalopathy with elevated ammonia develop. Liver biopsy is not required for diagnosis. Liver biopsy typically shows microvesicular fatty infiltration in the hepatocytes [12]. Imaging studies are also of little value for diagnosis of AFLP [10] and are useful to rule out conditions such as hepatic ischemia, hepatic infarct, Budd–Chiari syndrome, or hepatic hematoma/rupture.

### 2.4. Management

Prompt delivery after initial support measures is a cure [11,12,13]. When complications are present, complete recovery may take days to weeks. Prothrombin time improvement is the first sign of hepatic recovery. In general, there is no hepatic sequela after recovery. Rarely, AFLP patients with severe and rapidly progressing acute hepatic failure are referred for liver transplantation.

## 3. Mitochondrial Dysfunction and Pathogenesis of AFLP

Although the pathogenesis of AFLP remains largely unknown, molecular advances in the past 2 decades strongly suggest that mitochondrial dysfunction plays an important role in the pathogenesis of AFLP [14]. There is a strong association between maternal AFLP and impaired fetal and placental fatty-acid oxidation. There is ample data that link deficiency of long-chain 3-hydroxyacyl-CoA dehydrogenase (LCHAD) in the fetus to development of AFLP in the mother. 

### 3.1. Mitochondrial Dynamics and Biogenesis

Mitochondria are double-layered membranous organelles with a mitochondrial outer membrane (MOM) separating intermembrane space from the cytoplasm and a mitochondrial inner membrane (MIM) separating the mitochondrial matrix from the intermembrane space [15]. Furthermore, mtDNA encodes 13 polypeptides of mitochondrial respiratory chain (MRC) complexes and adenosine triphosphate (ATP) synthase, along with 22 transfer RNAs and 2 ribosomal RNAs encoded by nuclear DNA required for intra-mitochondrial translation. Mitochondria are also known as the cell’s “powerhouse” because they are the primary source of ATP production using substrates derived from lipid or glucose. Each hepatocyte contains about 800 mitochondria, accounting for around 18% of the total volume of the liver cell. Mitochondrial DNA is extremely vulnerable to oxidative damage due to the incomplete DNA repair mechanisms in mitochondria. 

Mitochondria are dynamic organelles that change their structure and shape in response to the energy demand and supply through fusion and fission processes [16,17]. Mitochondrial fission and fusion play important roles in maintaining the function of mitochondria under conditions of metabolic or environmental distress. Fusion is a process that helps mitigate cellular stress by mixing the contents of partially damaged mitochondria to promote complementation. Fission is needed to create new mitochondria, but it also contributes to quality control by enabling the removal of damaged mitochondria. Disruptions of these processes have been implicated in disease. Mitochondrial fission occurs when oxidative stress damages mitochondria, resulting in the separation of damaged mitochondria from healthy mitochondria [18,19]. Mitochondrial fusion-fission balance is disrupted by intracellular and external stress, resulting in mitochondrial fragmentation [20]. Mitochondrial dysfunction is associated with excessive fission, characterized by increased levels of the fission protein dynamin-related protein 1 (Drp1). The dysregulation of proteins involved in mitochondrial fission has an important impact on mitochondrial morphology and function [21,22]. In mammalian cells, mitofusin-1 (Mfn1) and mitofusin-2 (Mfn2) are the primary mediators of mitochondrial fusion and are responsible for the fusion process of the outer mitochondrial membrane (OMM) [23]. The protein Optic atrophy 1 (Opa1) regulates the fusion of the inner mitochondrial membrane (IMM), which is required for maintaining the balance between mitochondrial fusion and fission [24,25]. In clinical conditions involving placental dysfunction, changes in the expression of OPA1, SIRT3, and MFN1 have been identified [26,27]. PGC-1α (peroxisome proliferator-activated receptor gamma co-activator 1) and nuclear respiratory factor 1 (NRF1), which regulate expression of mtDNA and nuclear DNA genes encoding subunits of the MRC complexes and mtDNA replication and transcription, respectively, control mitochondrial biogenesis [28,29]. Because mitochondria are essential for metabolism and energy production [30,31,32] as well as regulating signaling pathways that mediate these processes [33,34], changes in mitochondrial dynamics can play an important role in the onset and progression of liver disease [35]. Mitophagy is a mechanism that involves the degradation of mitochondria. Mitophagy is essential for mitochondrial and cellular homeostasis during hepatic stress, and decreased mitophagy has been linked to mitochondrial dysfunction [36,37,38]. Mitophagy regulates liver metabolism and protects mitochondrial bioenergetics, preventing cell death and reducing oxidative stress [32]. Mitophagy induction is dependent on mitochondrial fission in liver hepatocytes. Excessive mitochondrial fission is a precursor to hepatocyte death since it causes mitochondrial dysfunction. Mitochondrial dysfunction in hepatocytes is often linked to metabolic abnormalities in fatty liver disease patients. The mitochondrial quality control (MQC) mechanism involves the complex regulation of biogenesis and mitophagy to maintain cellular homeostasis [39,40].

### 3.2. Mitochondrial Fatty-Acid Oxidation (FAO)

#### 3.2.1. Fatty Acid Transport to Mitochondria

**Figure 1:**
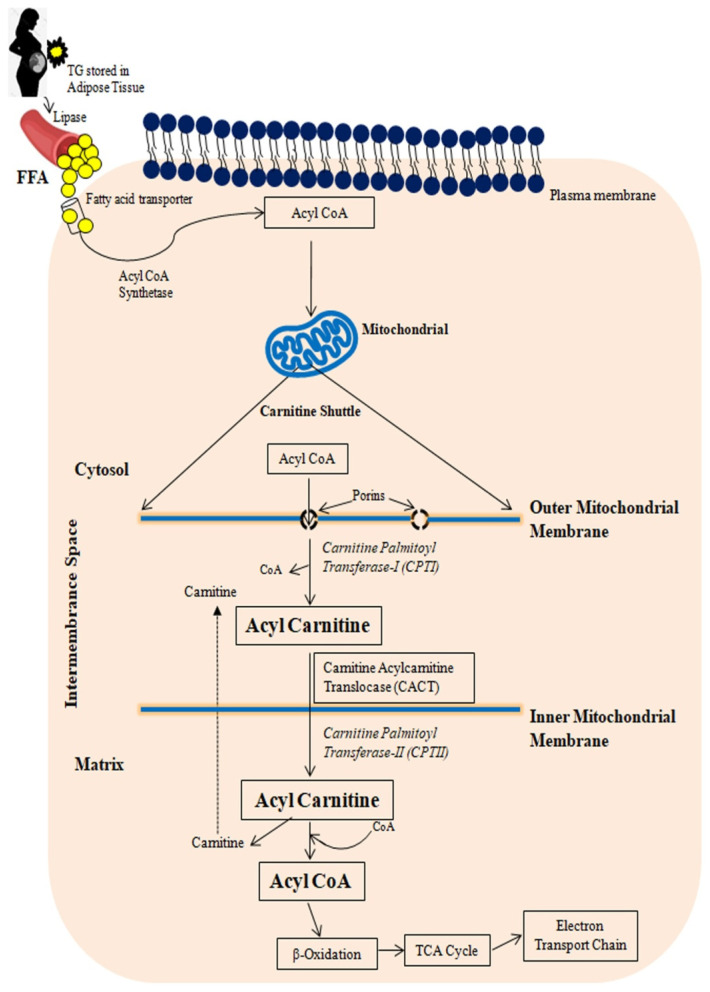
A schematic representation of free fatty acid transport to mitochondria. TG: Triglyceride; FFA: Free fatty acid.

Figure 1 provides a schematic representation of FA transport to mitochondria. Free fatty acids (FFAs) in the liver are derived from plasma FFAs produced by adipose tissue and chylomicrons, or they are synthesized de novo in the liver. These FFAs are either oxidized in mitochondria or esterified as triglycerides in hepatocytes, where they accumulate as fat droplets or are packaged with apolipoprotein B, cholesterol esters, and phospholipids to be secreted as very-low-density lipoproteins (VLDLs) [32,41].

De novo lipogenesis (DNL) is the mechanism of synthesizing lipids from either exogenous or endogenous energy sources. The major steps involved are FA synthesis from acetyl-CoA subunits formed during glycolysis and carbohydrate metabolism, FA elongation and desaturation form long-chain unsaturated FAs, and FA assembly into TGs and VLDLs [42].

Fatty acids are transported to the cell’s cytoplasm via a fatty acid transporter. Until joining mitochondria, FFAs must be converted to Acyl CoA by the Fatty Acyl Synthase (FAS), a multi-enzymatic complex. Carnitine shuttle transports acyl-CoA to the mitochondria. An acyl-CoA must first be transported via the outer and inner mitochondrial membranes using the carnitine palmitoyl transferase transport mechanism before being β-oxidized in the mitochondrial matrix. Acyl binds to carnitine through the mitochondrial outer membrane enzyme carnitine palmitoyl transferase I (CPTI). The acyl-carnitine molecule is then transported through the mitochondrial inner membrane by the acyl-carnitine translocase (CACT). Carnitine palmitoyl transferase II (CPTII) separates acyl from carnitine in the mitochondrial matrix, allowing for the formation of acyl-CoA. The acyl-carnitine translocase then releases the carnitine into the intermembrane space, where it is used again [43]. Until the final cleavage, one of the acyl-CoA-dehydrogenases transforms the acyl-CoA-ester into a trans-2-enoyl-CoA, which is then hydroxylated into β-hydroxyacyl-CoA and dehydrogenated into 3-keto-acyl-CoA. The tricarboxylic acid cycle can use the acetyl-CoA formed, and the reducing agents transport the electrons to the electron transport chain [44]. 

#### 3.2.2. Mitochondrial β-Oxidation Cycle

**Figure 2:**
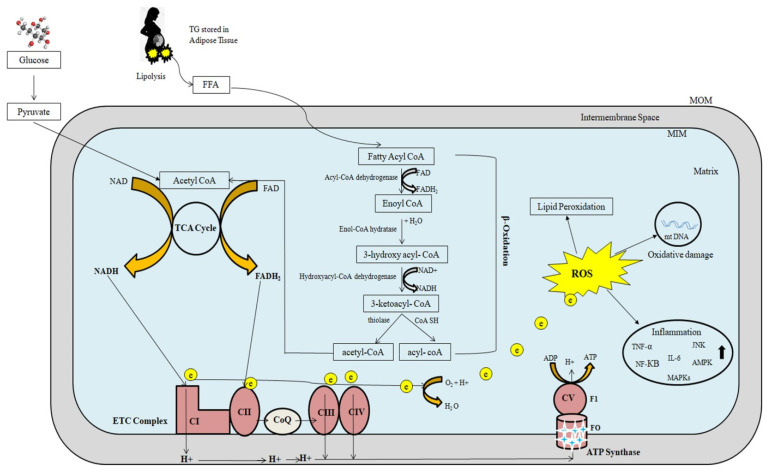
The role of mitochondria in fatty-acid oxidation and energy production. Excessive electron leakage causes excessive ROS generation and cellular injury. FFA: Free fatty acid; NADH: Nicotinamide adenine dinucleotide; FADH: Flavin adenine dinucleotide; ROS: Reactive oxygen species; ETC: Electron transport chain; MOM: Mitochondrial outer membrane; MIM: Mitochondrial inner membrane; ADP: Adenosine diphosphate; e: electrons; ATP: Adenosine triphosphate; TNF-α: Tumor necrosis factor-α; NF-KB: Nuclear factor-KB; IL: Interleukin; MAPKs: Mitogen activated protein kinases; JNK: c-jun N terminal kinase; AMPK: AMP-activated protein kinase.

Figure 2 depicts a schematic representation of mitochondrial fatty-acid oxidation and energy production. Mitochondrial β-oxidation is the primary oxidative mechanism for fatty acids including the oxidation of short-chain (<C6), medium-chain (C6–C12), and long-chain (>C12) fatty acids [45]. Long-chain fatty acids can only reach the mitochondria via CPT1 transport mechanism [46]. Short- and medium-chain fatty acids can freely enter the mitochondria. As a result, CPT1 in the mitochondria is the rate-limiting enzyme in β-oxidation.

Three types of acyl-CoA dehydrogenases are known to play roles in FAO in humans: very-long-chain, medium-chain, and short-chain acyl-CoA dehydrogenases (VLCAD, MCAD, and SCAD, respectively). These three enzymes work together to convert long-chain acyl-CoAs to medium-chain acyl-CoAs and then to short-chain acyl-CoAs. MCAD and SCAD are soluble, matrix-localized enzymes, while VLCAD is associated with the inner mitochondrial membrane. Mitochondrial trifunctional protein (MTP) is a complex enzyme that contains three enzymatic activities: long-chain enoyl-CoA hydratase, 3-hydroxyacyl-CoA dehydrogenase, and 3-ketothiolase. The α-subunit contains long-chain enoyl-CoA hydratase (LCEH) and long-chain 3-hydroxyacyl-CoA dehydrogenase (LCHAD), while the β-subunit contains long-chain 3-ketoacyl-CoA thiolase (LCKAT). MTP is bound to the inner mitochondrial membrane. The matrix-localized enzymes MCAD, crotonase (enoyl-CoA hydratase), MCHAD, and MCKAT manage the resulting medium-chain acyl-CoAs. Finally, SCAD, crotonase, SCHAD, and MCKAT metabolize short-chain acyl-CoAs.

#### 3.2.3. Oxidative Phosphorylation

The main source of cellular energy is the electron transport chain (ETC) and oxidative phosphorylation through critical activities of protein complexes in the inner mitochondrial membrane. High-energy electrons released during the citric acid cycle and β-oxidation are captured by nicotinamide adenine dinucleotide (NAD) and flavin adenine dinucleotide (FAD), resulting in NADH and FADH_2_, respectively [47]. NADH and FADH_2_ molecules donate these high-energy electrons to the ETC [48]. The transfer of electrons to O_2_ is an energy-yielding reaction by the passage of electrons through a series of carriers, which constitute the ETC. These carriers include four complexes (complex I, II, III, IV) in the inner mitochondrial membrane. A fifth protein complex (complex V), also in the inner mitochondrial membrane, then serves to couple the energy-yielding reactions of electron transport to ATP synthesis. Complex I receives electrons from NADH, while complex II receives electrons from FADH_2_. Complexes I and II provide electrons to Coenzyme Q (CoQ). CoQ (also called ubiquinone) is a small, lipid-soluble molecule that carries electrons through the MIM to complex III. Electrons are then transferred from complex III to cytochrome *c*, which then carries electrons to complex IV (cytochrome oxidase), where they are finally transferred to O_2_. Water is formed as a result of electron transfer from Complex IV to oxygen. At complexes I, III, and IV, free energy is released as electrons pass along the chain, which is utilized to pump protons from the mitochondrial matrix to the intermembranous region, forming a proton gradient. The potential energy stored in this gradient is then used by a fifth protein complex (complex V), which couples the flow of protons along the electrochemical gradient back across the MIM to the synthesis of ATP. This process is depicted in Figure 2. ETC malfunction can cause an excessive amount of electron leak, resulting in an excessive amount of ROS production and cellular damage (Figure 2).

### 3.3. Regulation of Mitochondrial Fatty-Acid Oxidation and Reactive Oxygen Species Formation

CPT1 is inhibited by malonyl-CoA, which is formed during the first step of the synthesis of FFAs from acetyl-CoA by acetyl-CoA carboxylase [32,35]. Insulin has been shown to increase malonyl-CoA synthesis, which inhibits CPTI. Glucagon, on the other hand, decreases malonyl-CoA synthesis, leading to an increase in β-oxidation [49]. FFAs are degraded into acetyl-CoA molecules, which can either be fully degraded to CO_2_ by the Krebs cycle or condensed into ketone bodies, which are re-oxidized in peripheral tissues during fasting [30,32]. Under normal circumstances, this process carefully controls energy storage and disposal; however, it is hampered in patients with fatty liver disease, causing oxidative stress [30,50,51]. Increased oxidative stress causes inflammation directly by activating a number of inflammatory-signaling pathways, including the NF-κB and JNK pathways, as well as indirectly by increasing the gene expression of inflammatory cytokines including TNF-α, TGF-β, and Fas ligand [52]. Reduced mitophagy leads to an accumulation of significantly damaged mitochondria, which causes cell necrosis and the release of mitochondrial damage-associated molecular patterns (DAMPs), which may promote liver inflammation [53].

ROS such as superoxide anions, peroxides, and others are generated in the cytosol by enzymes such as amino acid oxidases, cyclooxygenases, lipoxygenases, nitric oxide (NO) synthase, and xanthine oxidase [54,55,56]. By transferring a single electron from NADPH to molecular oxygen, it becomes NADPH oxidase, which is the key source of ROS in liver diseases and produces superoxide anions in the mitochondria [57,58,59]. Recently, emerging evidence suggests that the flavin mononucleotide group of complex I is the main site of superoxide generation in the mitochondria through reverse electron transfer, which is consistent with data showing that diphenyleneiodonium inhibits succinate-related ROS generation without affecting the flavin group of complex II [60,61,62]. Furthermore, the ubiquinone-reactive sites Q_0_ and Q_i_ in complex III of the mitochondrial respiratory chain produce ROS species [63,64]. Electron leakage from NADPH to p450 within the microsomal monooxygenase (MMO) system, caused by a low efficiency of coupling, plays a critical role in ROS generation in hepatocytes [65].

Mitochondrial ROS activate AMPK [33,35] and mitogen-activated protein kinases (MAPKs), including c-Jun N-terminal kinase (JNK) [66]. AMPK induces PGC-1α and promotes glucose and fatty-acid oxidation. PGC-1α interacts with the peroxisome proliferator–activated receptor (PPAR) to increase mitochondrial fatty acid β-oxidation by inducing the expression of multiple fatty acid-metabolizing enzymes, such as CPT1 and acyl-CoA dehydrogenases [67]. By activating NRF2, H_2_O_2_ production by mitochondria activates AMPK, which regulates antioxidant enzyme expression [35]. Proinflammatory cytokines such as interleukin 6 (IL-6), tumor necrosis factor (TNF-α), and interleukin 1β (IL-1β) are also stimulated by ROS development. The presence of oxidative stress in cells may set off a chain reaction that contributes to increased mtDNA damage and increased mitochondrial dysfunction [68].

### 3.4. Mitochondrial Fatty-Acid Oxidation Defects

The last three steps of long-chain fatty-acid oxidation are catabolized by MTP, a heterooctamer of 4 α- and 4 β- subunits associated with the inner mitochondrial membrane [69]. The long-chain 3-enoyl-CoA hydratase enzymatic activity resides in the α-subunit amino-terminal domain while the carboxy-terminal domain contains the LCHAD enzymatic activity. The long-chain 3-ketoacyl-CoA thiolase enzymatic activity resides in the β-subunit. Both MTP subunit genes, *HADHA* and *HADHB*, are localized to chromosome 2p23 [70], and share a bidirectional promoter [71]. MTP defects are recessively inherited and can manifest as either an isolated LCHAD deficiency or complete MTP deficiency, in which all three enzymes are deficient [72]. Infants born with these recessively inherited disorders typically present with nonketotic hypoglycemia and hepatic encephalopathy, which may progress to coma and death [73]. They can also present as unexpected death, cardiomyopathy, or slowly progressive myopathy and peripheral neuropathy [74,75]. A common mutation in exon 15 of the α-subunit, G1528C, which causes an amino acid change at position 474 in the LCHAD catalytic site from glutamic acid to glutamine (E474Q) [76,77]. Our laboratory reported the α-subunit mutations in 24 patients with MTP defects [72]. Nineteen of the twenty-four patients had isolated LCHAD deficiency and presented with hepatic manifestations while the remaining five had complete MTP deficiency and presented with cardiac manifestations or a neuromuscular phenotype.

## 4. Fetal Mitochondrial Trifunctional Protein Defects and AFLP

Shoeman and colleagues were the first to describe in a case report a potential link between maternal AFLP and fatty-acid oxidation disorder in two siblings who both died at 6 months of age [78]. Subsequently, few other case reports have described an association between pediatric LCHAD deficiency and maternal liver disease [76,79,80]. Ibdah and colleagues reviewed the pediatric and maternal history in 24 families with documented MTP enzymatic deficiency and reported that 15 of the 24 mothers (62%) were diagnosed with AFLP, while the remaining nine women had normal pregnancies [72]. The molecular analysis revealed that affected infants did not have the G1528C mutation in five of the normal pregnancies but rather other mutations that cause complete MTP deficiency. The remaining four normal pregnancies were associated with pediatric LCHAD deficiency. Thus 79% (15/19) of pregnancies with pediatric LCHAD deficiency were complicated by AFLP, whereas none of the pregnant mothers carrying fetuses with complete MTP deficiency developed maternal liver disease [72]. This study suggested that a woman who carries a fetus with LCHAD deficiency has a 79% probability that her pregnancy will be complicated by maternal AFLP. In a follow up study, Ibdah and colleagues reported fetal genotypes and pregnancy outcomes in 83 pregnancies in 35 families with documented pediatric MTP defects [81]. This study provided a clear link between fetal LCHAD deficiency and maternal AFLP.

Subsequently, Yang and Ibdah et al. prospectively screened for fetal MTP mutations in 27 pregnancies complicated by AFLP to assess the significance of the association between maternal AFLP and fetal MTP defects [82]. This study was based solely on the maternal history, and molecular testing was performed at birth. The results showed that out of the 27 pregnancies complicated by AFLP, 5 carried fetuses with MTP mutations. In all fetal genotypes, the G1528C mutation was present on one or both alleles. Thus, in approximately one of five pregnancies complicated by AFLP, the fetus is LCHAD-deficient; hence, it was strongly recommended that newborns in pregnancies complicated by AFLP be tested for the G1528C mutation. Based on these results, current guidelines for management of AFLP by American College of Gastroenterology (ACG) and American Association for the Study of Liver Diseases (AASLD) recommend molecular testing in the newborn for LCHAD deficiency [83,84]. This testing, when performed early after birth, can be lifesaving to the newborn as it may identify pediatric LCHAD-deficiency before manifesting the disease. LCHAD deficiency can be treated by early substitution of the long chain fatty acids with medium chain fatty acids and institution of a diet high in carbohydrate and low in fat [85]. Further, screening for MTP mutations in neonates of pregnancies complicated by AFLP allows genetic counseling for future pregnancies. Prenatal diagnosis utilizing Chorionic villus sampling can be performed to identify subsequent pregnancies at risk to be complicated by AFLP [86]. 

It should be noted that there are few case reports in the literature that suggest a potential association between AFLP and fetal FAO disorders other than LCHAD deficiency [87,88]. Further investigation is warranted to understand the role of other FAO oxidation disorders in development of AFLP and the likely mechanism of this potential association. 

## 5. Mechanism of the Association between Fetal LCHAD Deficiency and AFLP 

**Figure 3:**
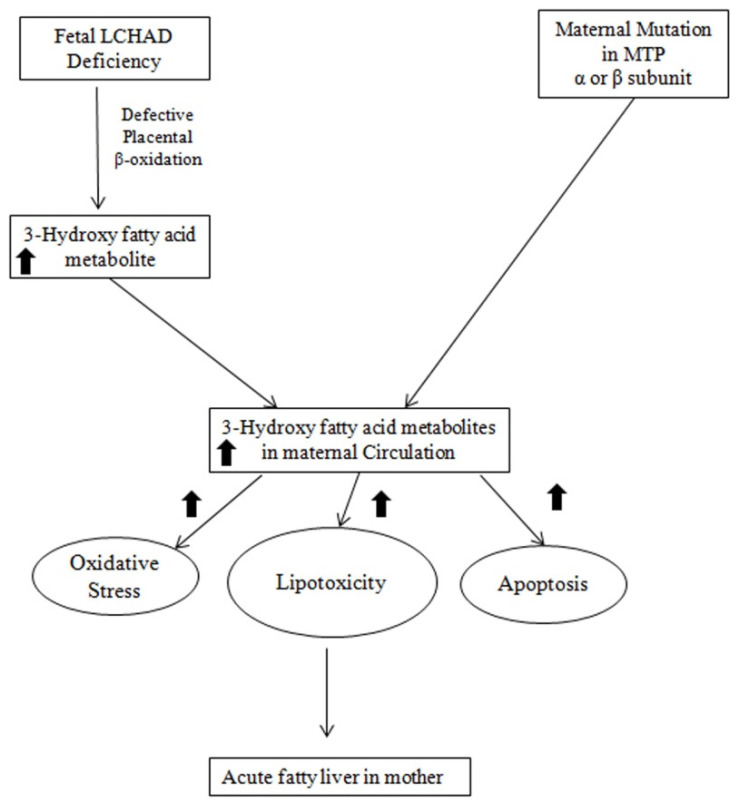
A schematic representation of possible mechanisms leading to the development of maternal AFLP associated with fetal LCHAD deficiency. MTP: Mitochondrial Trifunctional Protein; LCHAD: Long chain acyl-CoA dehydrogenase.

The precise mechanism for the association between fetal LCHAD-deficiency and maternal AFLP is not fully elucidated. Figure 3 depicts the likely mechanisms underlying the association between fetal LCHAD and maternal AFLP. Mitochondrial dysfunction and damage have been documented in children with LCHAD deficiency [89,90,91]. It is likely that hepatotoxic long-chain 3-hydroxyacyl fatty acid intermediates produced in the fetus due to blockages in the mitochondrial β-oxidation caused by the fetal LCHAD deficiency will accumulate in the maternal circulation causing liver injury and AFLP. It is also highly likely that the placenta is the major source for the 3-hydroxy fatty acid metabolites. This is supported by several studies that have shown high levels of FAO activity and increased expression of FAO enzymes in the placenta compared to the liver [92,93,94,95]. Furthermore, placental injury was reported in women who carry fetuses with LCHAD deficiency [89]. Placenta from patients with maternal AFLP were also reported to have an increase in placental lipid droplet accumulation and lipotoxicity [96]. In addition, maternal factors are likely to contribute to the accumulation of 3-hydroxy metabolites in the maternal circulation: First, the heterozygous mother has reduced capacity to oxidize long chain fatty acids. Second, the third trimester in pregnancy is associated with increased lipolysis and decreased β-oxidation.

The accumulation of cytotoxic 3-hydroxy fatty acids in maternal circulation is likely to cause microvesicular steatosis in maternal liver with disruption of β-oxidation and oxidative phosphorylation processes in the liver, causing decreased ATP production and increased ROS, leading to further mitochondrial damage [97,98,99]. Damage to the mitochondrial membrane in the liver was reported in a rat model of microvesicular steatosis [100,101]. Increased superoxide generation associated with reduced respiration and alterations to mitochondrial calcium homeostasis were also reported in placenta isolated from patients with AFLP. Furthermore, placental mitochondria in patients with AFLP demonstrate increased oxidative injury biomarkers [102,103]. More supporting evidence of increased oxidative stress in maternal AFLP is the reduced circulating levels of antioxidants such as tocopherols and retinol in patients with AFLP [100]. It is also possible that oxidative stress reduces the stability of *HADHA* protein in the mitochondrion. Further studies are needed to examine the effects of oxidative stress on *HADHA* protein stabilization. 

In addition to 3-hydorxy fatty acid intermediates, long-chain fatty acids such as palmitic acid and arachidonic acid are also elevated in the maternal circulation of patients with AFLP. There is supporting evidence that long-chain-fatty-acid accumulation can induce hepatocyte lipoapoptosis [100,103]. Caspase-dependent hepatocyte lipoapoptosis was reported to be induced by saturated-free, long-chain fatty acids such as palmitate [104,105]. In addition, there is evidence that palmitate-induced lipoapoptosis occurs via the activation of mitogen-activated protein kinase (MAPK) and forkhead family of transcription factor class O3 (FoxO3) and its downstream targets such as p53-upregulated modulator of apoptosis (PUMA) protein and pro-apoptotic microRNA 34a [104,106,107]. 

## 6. Conclusions

In conclusion, AFLP is a rare but life-threatening complication of pregnancy with serious fetal and maternal consequences. The pathogenesis of AFLP is strongly linked to mitochondrial dysfunction associated with fetal LCHAD deficiency. Current evidence supports an important role for placental injury and oxidative stress causing subcellular damage and mitochondrial dysfunction. The release of toxic 3-hydroxy intermediate metabolites from the LCHAD-deficient placenta and fetus into the maternal circulation is likely to be a culprit in inducing AFLP in the pregnant mother. Further research is needed to explore the role of 3-hydroxy fatty acid metabolites in the pathogenesis of AFLP.
